# Mother’s education and the risk of several neonatal outcomes: an evidence from an Italian population-based study

**DOI:** 10.1186/s12884-017-1418-1

**Published:** 2017-07-12

**Authors:** Anna Cantarutti, Matteo Franchi, Matteo Monzio Compagnoni, Luca Merlino, Giovanni Corrao

**Affiliations:** 1 Department of Statistics and Quantitative Methods, Division of Biostatistics, Epidemiology and Public Health, Laboratory of Healthcare Research and Pharmacoepidemiology, University of Milano-Bicocca, Via Bicocca degli Arcimboldi 8, U7, 20126 Milan, Italy; 2 Operative Unit of Territorial Health Services, Region Lombardia, Milan, Italy

**Keywords:** Socioeconomic inequality, Maternal education, Maternal birthplace, Adverse neonatal outcomes, Pregnancy and birth

## Abstract

**Background:**

Maternal socioeconomic disparities strongly affect child health, particularly in low and middle income countries. We assessed whether neonatal outcomes varied by maternal education in a setting where healthcare system provides universal coverage of health services to all women, irrespective of their socioeconomic status.

**Methods:**

A population-based study was performed on 383,103 singleton live births occurring from 2005 to 2010 in Lombardy, an Italian region with approximately 10 million inhabitants. The association between maternal education, birthplace and selected neonatal outcomes (preterm birth, low birth weight, small-for-gestational age, low 5-min Apgar score, severe congenital anomalies, cerebral distress and respiratory distress) was estimated by fitting logistic regression models. Model adjustments were applied for sociodemographic, reproductive and medical maternal traits.

**Results:**

Compared with low-level educated mothers, those with high education had reduced odds of preterm birth (Odds Ratio; OR = 0.81, 95% CI 0.77–0.85), low birth weight (OR = 0.78, 95% CI 0.70–0.81), small for gestational age (OR = 0.82, 95% CI 0.79–0.85), and respiratory distress (OR = 0.84, 95% CI 0.80–0.88).

Mothers born in a foreign country had higher odds of preterm birth (OR = 1.16, 95% CI 1.11–1.20), low Apgar score (OR = 1.18, 95% CI 1.07–1.30) and respiratory distress (OR = 1.19, 95% CI 1.15–1.24) than Italian-born mothers. The influence of maternal education on neonatal outcomes was confirmed among both, Italian-born and foreign-born mothers.

**Conclusions:**

Low levels of education and maternal birthplace are important factors associated with adverse neonatal outcomes in Italy. Future studies are encouraged to investigate factors mediating the effects of socioeconomic inequality for identifying the main target groups for interventions.

**Electronic supplementary material:**

The online version of this article (doi:10.1186/s12884-017-1418-1) contains supplementary material, which is available to authorized users.

## Background

Maternal socioeconomic status (SES) strongly affects child health [1–6], likely attributed to delayed prenatal care, preterm delivery and adverse birth outcomes [7–14]. Different SES measures capture unique aspects and pathways of socioeconomic disparities that can relate differently to child health. For example, maternal education reflects life-course SES [15], including parents’ SES during childhood and adolescence, access to higher education, work opportunities, and income during adulthood [16]. According to a systematic review of studies in industrialized countries, maternal education, rather than maternal income, has been found to correlate with birth outcomes [17].

Differences in the ability to access good-quality obstetric services and neonatal care may be due to differences in maternal socioeconomic status [2]. The Italian National Health Service (NHS) provides universal coverage for many areas of healthcare, including obstetric, neonatal and related health care services to women, regardless of their SES [18]. Neonatal outcomes are expected to be only partially affected by socioeconomic inequalities in health systems with universal access to essential health services [2].

We performed a large population-based study aimed to measure the relationship between maternal education and several neonatal outcomes (i.e., preterm birth, low birth weight, small for gestational age, 5-min Apgar less than 7, severe congenital anomalies, signs of cerebral distress and distress of respiratory functions) in the Italian region of Lombardy. Our analysis took into consideration other maternal features (i.e., maternal birthplace, sociodemographic factors, reproductive history, and medical conditions), as well as investigating the impact of all maternal traits.

## Methods

### Setting

Data obtained for this study were retrieved from the healthcare utilization (HCU) databases of Lombardy, a region of Italy which accounts for approximately 16% (~ 10 million) of the national population. In Italy, the entire population is covered by the NHS, which in Lombardy has been active since 1997 with an automated system of databases to collect a variety of HCU information. For the purpose of the current study, the following databases were considered: (i) the archive of beneficiaries of the Regional Health Service (RHS), i.e., the entire resident population, reporting demographic and administrative data (e.g., municipality, date of birth and date of start and end of being RHS beneficiary), (ii) the database on diagnosis at discharge from public or private hospitals of Italy (diagnoses classified according to the International Code of Disease, 9th Revision, ICD-9); and (iii) the database reporting Certificates of Delivery Assistance (CeDAP) including information self-reported by the mother relating to her socioeconomic traits in the period recent to her current pregnancy, other than medical information relating to pregnancy, childbirth, and child presentation at delivery. In general, information was collected and directly added to the specific database when the specific service was provided, for example, when an individual was recorded for being a RHS beneficiary, a patient discharged from hospital, or a woman who gave birth.

As each single record for the aforementioned databases utilises an univocal identification code, the record linkage between databases was allowed. In order to preserve privacy, however, each identification code was automatically converted into an anonymous code and the inverse process was prevented by the deletion of the conversion table. For the current application, a deterministic procedure of record linkage between the above listed databases was performed so as to select the study cohort and collect data on maternal traits and newborn outcomes.

### Cohort selection

**Fig. 1:**
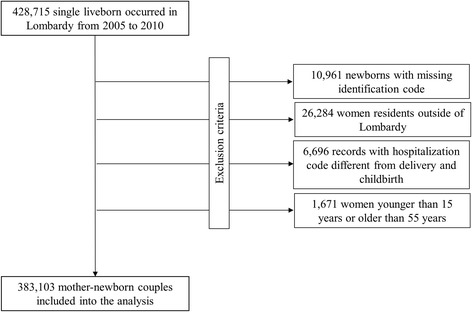
Flow-chart of inclusion and exclusion criteria

The 428,715 singleton live births that occurred in Lombardy from 2005 to 2010 were selected from the CeDAP database, provided that identification codes of both mother and newborn were reported. We sequentially excluded (Fig. 1) (i) 10,961 newborns (2.6%) because of a missing identification code (CeDAP database); (ii) 26,284 records (6.3%) because the mother was resident outside the Lombardy region (RHS beneficiaries archive); (iii) 6696 records (1.7%) because the reported hospital admission ICD-9 code of mother and/or newborn was different from that of the delivery and/or birth (hospital discharge database); and (iv) 1671 records (0.4%) because the mother was younger than 15 years or older than 55 years of age at delivery (RHS beneficiaries archive). The final study cohort included 383,103 mother-newborn couples.

### Collection of data on maternal traits

Information on maternal traits at the time of delivery was obtained from the CeDAP database and included age at delivery (≤25, 25–34 and ≥35 years), sociodemographic factors and reproductive history. Sociodemographic factors included (i) education, measured according to the length of formal education completed and categorized as ≤8 years (low), from 9 to 13 years (intermediate), and ≥14 years (high); (ii) birthplace, categorized as Italian-born and foreign-born, (iii) employment, categorized as employed and unemployed (the latter including women without a job, housewives and students); and (iv) marital status, categorized as married and unmarried. Reproductive history included (i) parity categorized as null parity and multi parity; and (ii) previous spontaneous miscarriages (yes/no). In addition, maternal medical conditions were identified from inpatient diagnoses (hospital discharge database) within the 2 years prior to date of delivery and included hypertension, dyslipidaemia, diabetes and preeclampsia. Additional file 1: Table S1 presents the ICD-9 codes used for identifying maternal medical conditions.

### Identification of newborn outcomes

Newborn outcomes appearing at presentation and within 2 years after birth were respectively identified from the CeDAP and the hospital discharge database. At presentation, we considered preterm birth (less than 37 weeks’ gestation [19]), low birth weight (below 2500 g [20]), small for gestational age (birth-weight less than 10th percentile for infants from 22 to 43 weeks [21, 22]), and low 5-min Apgar score (5-min Apgar <7 [23]).

From the hospital discharge database the following three categories of neonatal outcomes were considered: (i) severe congenital anomalies, defined according to the EUROCAT classification (www.eurocat-network.eu) and included anomalies of the nervous, respiratory, digestive, urinary and genital systems, and defects of eye, ear, face and neck, heart, abdominal wall and limb; (ii) cerebral distress, including convulsion, other and unspecified cerebral irritability in newborn, cerebral depression, coma, and other abnormal cerebral signs; and (iii) distress of respiratory function, including intrauterine hypoxia, birth asphyxia and other respiratory conditions of foetus and newborn. Additional file 1: Table S2 summarises ICD-9 codes used for identifying these categories of newborn outcomes. Primary or secondary diagnosis were considered for identifying the onset of outcome.

### Statistical analysis

The frequency of a given neonatal outcome within strata of the considered maternal traits was evaluated by testing for heterogeneity between strata (of maternal birthplace, employment, marital status, reproductive history and medical conditions) or trend over strata (of educational status and age at delivery) respectively according to chi-square test, or its version for trend.

A logistic regression model was fitted to estimate the odds ratio (OR), and its 95% confidence interval (CI), of a given neonatal outcome in relation to categories of maternal education and birthplace. The influence of maternal education on neonatal outcomes was evaluated by considering the entire sample of mother-newborn couples in addition to stratifying data according to maternal birthplace. Linear trend in ORs for different levels of education was tested by using the contrast statement implemented in SAS [24]. Model adjustments were made for the above reported sociodemographic, reproductive and medical maternal traits.

The following two expedients were used for taking into account the nature of our data. First, because of the potential correlation of women contributing to more than one birth during the considered period, the models were fitted using Generalized Estimating Equations (GEE) for correlated observations with a logit link [25]. Two, because data were missing for some women (ranging missing values from 1% for previous miscarriages to 13% for marital status), 100 multiple imputations were applied by using the fully conditional specification (FCS) method implemented in SAS [26, 27].

All analyses were performed using the Statistical Analysis System Software (version 9.4; SAS Institute, Cary, NC, USA). Statistical significance was set at the 0.05 level. All *p*-values were two-sided.

## Results

Just over 1 in 20 newborns were found to be affected from low birth weight (prevalence 5.1%), respiratory distress (5.1%), preterm birth (5.3%), small for gestational age (7.8%) and severe congenital anomalies (5.0%). Lower prevalence was observed for low Apgar score (0.8%) and cerebral distress (0.3%).

It also emerged that as educational level increases, the frequency of several outcomes (i.e., preterm birth, low birth weight, small for gestational age, cerebral distress and respiratory distress) decreases proportionally (Table 1). Other maternal traits (e.g., older age, foreign-born, unmarried and unemployment status, null parity, previous miscarriages and suffering from medical conditions) were significantly associated with several neonatal outcomes.

**Table 1:** Frequency of neonatal outcomes according to selected maternal traits. Italy, Lombardy Region, 2005–2010

	All women	Preterm birth	Low birth weight	Small for Gestational Age	Low Apgar score	Congenital Anomalies	Cerebral distress	Respiratory distress
	(*N* = 383,103)	(*N* = 20,294)	(*N* = 19,588)	(*N* = 29,800)	(*N* = 3180)	(*N* = 18,997)	(*N* = 996)	(*N* = 15,539)
Maternal trait	%	%	%	%	%	%	%	%
Education^a^								
Low	121,910	5.8%	5.6%	8.1%	0.9%	4.9%	0.3%	5.4%
Intermediate	173,926	5.2%	5.1%	7.7%	0.8%	4.8%	0.3%	5.0%
High	87,267	4.7%	4.6%	7.3%	0.7%	5.2%	0.2%	4.7%
*p*-value^b^		<0.0001	<0.0001	<0.0001	0.0466	0.0044	0.0444	<.0001
Maternal birthplace								
Italian-born	288,093	5.2%	4.9%	8.1%	0.8%	4.9%	0.2%	4.9%
Foreign-born	95,010	5.6%	5.2%	6.8%	0.9%	4.9%	0.3%	5.6%
*p*-value^b^		<0.0001	0.0009	<0.0001	0.0001	0.1617	0.0200	<.0001
Age at delivery								
≤ 25 years	49,803	4.9%	4.9%	8.4%	0.9%	4.8%	0.2%	5.2%
26–35 years	244,037	5.0%	4.9%	7.8%	0.8%	4.8%	0.3%	4.9%
≥ 35 years	89,263	6.2%	5.8%	7.5%	0.9%	5.5%	0.2%	5.4%
*p*-value^b^		<0.0001	<0.0001	0.1739	0.0009	<.0001	0.3189	<.0001
Marital status								
Married	294,606	5.2%	4.9%	7.4%	0.8%	4.9%	0.3%	4.9%
Unmarried	88,497	5.8%	5.9%	9.1%	0.9%	5.1%	0.3%	5.6%
*p*-value^b^		<0.0001	<0.0001	<0.0001	0.0003	0.0245	0.7656	<.0001
Employment								
Employed	270,088	5.2%	5.1%	7.9%	0.8%	5.0%	0.2%	5.0%
Unemployed	113,015	5.4%	5.1%	7.5%	0.9%	4.8%	0.3%	5.2%
*p*-value^b^		0.0170	0.3370	0.0003	0.1516	0.0691	0.0454	0.0068
Parity								
Nulliparous	211,090	5.7%	6.0%	9.7%	0.9%	5.4%	0.3%	5.9%
Multiparous	172,013	4.8%	3.9%	5.5%	0.7%	4.5%	0.2%	4.0%
*p*-value^b^		<0.0001	<0.0001	<0.0001	<0.0001	<0.0001	<.0001	<.0001
Previous spontaneous abortions								
No	320,274	5.1%	5.0%	7.9%	0.8%	4.9%	0.3%	5.1%
Yes	62,829	6.1%	5.6%	7.0%	0.9%	5.2%	0.3%	4.9%
*p*-value^b^		<0.0001	<0.0001	<0.0001	0.0689	0.0008	0.5183	0.0223
Diabetes								
No	371,227	5.2%	5.1%	7.8%	0.8%	4.9%	0.3%	5.0%
Yes	11,915	9.3%	5.8%	6.1%	1.4%	6.5%	0.4%	6.8%
*p*-value^b^		<0.0001	0.0007	<0.0001	<0.0001	<0.0001	0.0019	<.0001
Hypertension								
No	370,077	5.0%	4.8%	7.6%	0.8%	4.9%	0.3%	5.0%
Yes	13,026	12.9%	14.6%	12.8%	1.4%	5.9%	0.3%	6.9%
*p*-value^b^		<0.0001	<0.0001	<0.0001	<0.0001	<0.0001	0.7711	<.0001
Dyslipidaemia								
No	382,202	5.3%	5.1%	7.8%	0.8%	4.9%	0.3%	5.1%
Yes	901	9.7%	6.9%	6.7%	0.9%	5.5%	0.5%	6.7%
*p*-value^b^		<0.0001	0.0158	0.2091	0.8481	0.4135	0.0817	0.0332
Preeclampsia								
No	373,909	4.8%	4.6%	7.5%	0.8%	4.9%	0.3%	4.9%
Yes	9194	26.0%	27.5%	17.7%	2.2%	7.9%	0.4%	11.5%
*p*-value^b^		<0.0001	<0.0001	<0.0001	<0.0001	<0.0001	0.0158	<.0001

^a^Years of formal education completed categorized as ≤8 years (low), from 9 to 13 years (intermediate), and ≥14 years (high)

^b^According to chi-square test or its version for the trend (education and age at delivery)

The relationship between maternal education and birthplace and selected neonatal outcomes is summarised in Table 2. With the exception of severe congenital anomalies, significant trends showing a decrease in adjusted ORs as maternal education increases were observed for all of the considered neonatal outcomes, including those recorded at presentation (preterm birth, low birth weight, small for gestational age), as well as those recorded within the first 2 years of life (cerebral distress and respiratory distress). Compared to Italian-born mothers, foreign-born mothers had a higher odds of preterm birth, low Apgar score and respiratory distress, while they had lower odds of being small for gestational age. The influence of maternal education on neonatal outcome was confirmed in both Italian-born and foreign-born mothers (Table 3).

**Table 2:** Relationship between maternal education and birthplace and selected neonatal outcomes. Italy, Lombardy Region, 2005–2010

	Preterm birth	Low birth weight	Small for Gestational Age	Low Apgar score	Severe congenital Anomalies	Cerebral distress	Respiratory distress
	OR ^b^ (95% CI)	OR ^b^ (95% CI)	OR ^b^ (95% CI)	OR ^b^ (95% CI)	OR ^b^ (95% CI)	OR ^b^ (95% CI)	OR ^b^ (95% CI)
Education ^a^							
Low	1.00 (reference)	1.00 (reference)	1.00 (reference)	1.00 (reference)	1.00 (reference)	1.00 (reference)	1.00 (reference)
Intermediate	0.90 (0.87–0.94)	0.87 (0.84–0.90)	0.88 (0.86–0.91)	0.98 (0.90–1.07)	0.94 (0.91–0.98)	1.00 (0.86–1.16)	0.91 (0.87–0.94)
High	0.81 (0.77–0.85)	0.78 (0.74–0.81)	0.82 (0.79–0.85)	0.92 (0.83–1.03)	1.02 (0.97–1.06)	0.84 (0.69–1.02)	0.84 (0.80–0.88)
p-trend ^b^	<0.0001	<0.0001	<0.0001	0.0164	0.1155	0.4745	<0.0001
Birthplace							
Italian-born	1.00 (reference)	1.00 (reference)	1.00 (reference)	1.00 (reference)	1.00 (reference)	1.00 (reference)	1.00 (reference)
Foreign-born	1.16 (1.11–1.20)	0.98 (0.94–1.03)	0.82 (0.79–0.85)	1.18 (1.07–1.30)	1.01 (0.97–1.06)	1.17 (0.99–1.39)	1.19 (1.15–1.24)

^a^ Years of formal education completed categorized as ≤8 years (low), from 9 to 13 years (intermediate), and ≥14 years (high)

^b^ Odds ratios (and 95% confidence interval) were derived from logistic regression. Full multivariable models for each outcome included as covariates maternal traits (i.e., age at delivery, marital status, employment, parity, previous spontaneous miscarriages, diabetes, hypertension, dyslipidaemia and preeclampsia) categorized as in Table 1

**Table 3:** Relationship between maternal education and selected neonatal outcomes according to maternal birthplace. Italy, Lombardy Region, 2005–2010

	Preterm birth	Low birth weight	Small for Gestational Age	Low Apgar score	Severe congenital Anomalies	Cerebral distress	Respiratory distress
	OR ^b^ (95% CI)	OR ^b^ (95% CI)	OR ^b^ (95% CI)	OR ^b^ (95% CI)	OR ^b^ (95% CI)	OR ^b^ (95% CI)	OR ^b^ (95% CI)
	Italian-born mothers						
Education ^a^							
Low	1.00 (reference)	1.00 (reference)	1.00 (reference)	1.00 (reference)	1.00 (reference)	1.00 (reference)	1.00 (reference)
Intermediate	0.88 (0.85–0.92)	0.86 (0.82–0.89)	0.88 (0.85–0.92)	0.95 (0.84–1.08)	0.97 (0.93–1.01)	0.99 (0.83–1.19)	0.90 (0.85–0.94)
High	0.79 (0.76 to 0.84)	0.77 (0.73 to 0.81)	0.82 (0.79 to 0.85)	0.98 (0.88–1.10)	1.06 (0.99–1.12)	0.85 (0.68–1.08)	0.84 (0.80–0.88)
p-trend ^b^	<0.0001	<0.0001	<0.0001	0.3129	0.0997	0.6704	<0.0001
	Foreign-born mothers						
Education ^a^							
Low	1.00 (reference)	1.00 (reference)	1.00 (reference)	1.00 (reference)	1.00 (reference)	1.00 (reference)	1.00 (reference)
Intermediate	0.94 (0.88–1.01)	0.90 (0.84–0.97)	0.88 (0.83–0.94)	0.97 (0.83–1.14)	0.88 (0.82–0.94)	1.00 (0.77–1.31)	0.92 (0.86–0.99)
High	0.84 (0.77–0.92)	0.78 (0.71–0.87)	0.84 (0.77–0.92)	0.81 (0.61–1.07)	0.90 (0.82–0.99)	0.78 (0.52–1.16)	0.81 (0.73–0.90)
p-trend ^b^	<0.0001	<0.0001	<0.0001	0.2615	<0.0001	0.9614	<0.0001

^a^ Years of formal education completed categorized as ≤8 years (low), from 9 to 13 years (intermediate), and ≥14 years (high)

^b^ Odds ratios (and 95% confidence interval) were derived from logistic regression. Full multivariable models for each outcome included as covariates maternal traits (i.e., age at delivery, marital status, employment, parity, previous spontaneous miscarriages, diabetes, hypertension, dyslipidaemia and preeclampsia) categorized as in Table 1

## Discussion

The main findings from the present study show that even in a country with universal access to essential health care services such as Italy, mothers with higher levels of education were at lower risk of several neonatal adverse outcomes. These differences cannot be underestimated, since compared to mothers with lower levels of education, those with high levels of education had 19, 22, 18, and 16% decreased risk of preterm birth, low birth weight, small for gestational age and respiratory distress, respectively. Corroborating our findings, a recent meta-analysis conducted across 12 European countries revealed a 48% risk excess of preterm births associated with low maternal education [28].

It was reported that among mother social aspects, education is considered the most powerful determinant of health [29]. Other mother’s traits influencing birth health, however, deserve to be mentioned. One, our study confirms previous observations that in Western countries a high proportion of births are to migrant women [30]. Migrant status has been associated with several adverse neonatal outcomes in some [31–36], but not all [36–41] studies, possibly because of differences in access to healthcare services [32, 42, 43], and integration policies of the host countries [44]. Our study shows that, compared to Italian-born mothers, foreign-born ones were at higher risk for preterm birth, low Apgar score and respiratory distress, while they had lower risk of being small for gestational age. Two, our study confirms that advanced maternal age [44–46], nulliparous [47], and unmarried status [48, 49] are risk factors for some adverse perinatal outcomes. Three, in the current study, unemployed mothers were at a higher risk of some adverse neonatal outcomes, likely because the condition might be a proxy of social inequality uncaptured by education and birthplace. This finding is consistent with studies showing the influence of employment status on preterm birth, small for gestational age and other neonatal outcomes [50, 51]. Finally, we confirmed previous evidence that diabetes, hypertension and to a greater extent pre-eclampsia and drug therapies for managing these concomitant diseases, are leading causes of adverse neonatal outcomes [52–57].

Our study has a number of potential limitations. First, the exclusion of mother-newborn pairs lacking identification codes could mainly affect less healthy women. Second, we did not collect information on income, a factor recognised to be associated with perinatal outcomes [1–4, 6]. More importantly, we did not have data on the country of origin of maternal birthplace. This may have resulted in residual confounding due to the unknown gradient of the effect of socioeconomic status. We are confident that the exclusion of this information did not influence the results observed since we also included information on maternal occupation. Third, privacy concerns did not allow of assessing the validity of information recorded in the Certificates of Delivery Assistance, as well as of diagnostic data from hospital charts. Finally, the lack of data on important factors, such as smoking, pre-pregnancy weight and gestational weight gain, may further contribute to some unavoidable source of systematic uncertainty.

## Conclusion

Notwithstanding these limitations, our study shows that, in a setting where healthcare system provides essential health services to all women, irrespective of their socioeconomic status, mother’s education and other socioeconomic factors are strongly associated with some adverse perinatal outcomes, including preterm birth, low Apgar score, cerebral distress, respiratory distress, and SGA. These findings merit attention from a public health perspective. Future studies are encouraged to investigate factors mediating the effects of socioeconomic inequality on birth outcomes for identifying the main target groups for interventions.

## Additional file

Additional file 1: The file includes the definition used to evaluate the presence of (i) the chronic maternal medical conditions (**Table S1**) and (ii) the neonatal outcomes considered (**Table S2**). (PDF 350 kb)

## Abbreviations

CeDAP: Certificates of Delivery Assistance
CI: Confidence interval
HCU: HealthCare Utilization
ICD-9: International code of disease, ninth revision
NHS: National Health Service
OR: Odds ratio
RHS: Regional Health Service
SES: Socioeconomic status
