# Supplementary material for: Mother’s education and the risk of several neonatal outcomes: an evidence from an Italian population-based study
Source: BMC Pregnancy Childbirth. 2017 Jul 12;17:221. doi: 10.1186/s12884-017-1418-1 (PMC5508478; doi:10.1186/s12884-017-1418-1)
Supplement: Additional file 1: — The file includes the definition used to evaluate the presence of (i) the chronic maternal medical conditions (Table S1) and (ii) the neonatal outcomes considered (Table S2). (PDF 350 kb) [file 12884_2017_1418_MOESM1_ESM.pdf]

Additional file

Table S1. Chronic maternal medical conditions were defined from inpatient diagnoses using ICD-9 code. They were measured from 2 years pre-LMP (last menstrual period) through to the end of the delivery.

**Condition**		**ICD-9 codes**
**Hypertension**	Pre-existing hypertension	401.x-405.x, 642.0x-642.2x, 642.7x, 642.9x
	Gestational hypertension	642.3x
**Preeclampsia**	Mild preeclampsia	642.4x
	Severe preeclampsia	642.5x, 642.6x, 642.7x
**Diabetes**	Pre-gestational diabetes	250.x, 648.0x
	Gestational diabetes	648.8x
**Dyslipidemia**	Disorders of lipid metabolism	272.x

Table S2. Neonatal outcomes within two years after birth, defined from hospital discharge database

Severe congenital anomalies – EUROCAT classification, www.eurocat-network.eu

Condition	ICD-9 code
Nervous system	740, 741, 742
Eye	743
Ear, face and neck	744
Congenital heart defects	745, 746, 747.0 – 747.4
Respiratory	748.0, 748.4, 748.50, 748.52, 748.58, 748.6, 748.8
Oro-facial clefts	749.0, 749.1, 749.2
Digestive system	750, 751, 756.6
Abdominal wall defects	756.71, 756.70, 756.79
Urinary	752.61, 753, 756.72
Genital	752.0 – 752.4, 752.60, 752.62, 752.7 – 752.9
Limb	754.3 – 754.8, 755

Signs of cerebral distress

Condition	ICD-9 code
Convulsions in newborn	779.0
Other and unspecified cerebral irritability in newborn	779.1
Cerebral depression, coma, and other abnormal cerebral signs	779.2

Distress of respiratory function

Condition	ICD-9 code
Intrauterine hypoxia and birth asphyxia	768
Other respiratory conditions of foetus and newborn	770
